# Cloning and Characterization of an Endolysin LysSA120 as a Potential *Staphylococcus* Biofilm-Removing Agent

**DOI:** 10.3390/v18060654

**Published:** 2026-06-09

**Authors:** Natalia N. Golosova, Yana A. Khlusevich, Bogdana I. Kravchuk, Lyudmila A. Emelyanova, Anna V. Sushko, Yulia N. Kozlova, Nina V. Tikunova, Andrey L. Matveev

**Affiliations:** Institute of Chemical Biology and Fundamental Medicine, Siberian Branch of the Russian Academy of Sciences, 630090 Novosibirsk, Russia; n.golosova@g.nsu.ru (N.N.G.); khlusevichjana@mail.ru (Y.A.K.); semali328@gmail.com (B.I.K.); mila.kuharenko@mail.ru (L.A.E.); a.sushko@g.nsu.ru (A.V.S.); ulona@ngs.ru (Y.N.K.); tikunova@niboch.nsc.ru (N.V.T.)

**Keywords:** antibiotic resistance, bacteriophage, endolysin, antimicrobial agent, *Staphylococcus aureus*, biofilm

## Abstract

*Staphylococcus aureus*, including methicillin- and vancomycin-resistant variants (MRSA, VRSA), causes infections that are increasingly difficult to treat with conventional antibiotics. One of the approaches to developing new therapeutics to treat staphylococcal infections is the use of bacteriophages specific to these bacteria or the lytic enzymes of such bacteriophages, which are capable of hydrolyzing the cell walls of these bacteria. Phage-encoded endolysins offer an alternative promising class of antimicrobial agents. In this study, LysSA120, a 250-amino-acid endolysin encoded by the *S. aureus* podophage vB_SauP_120, was cloned, expressed in *Escherichia coli*, and characterized. The domain organization and tertiary structure of LysSA120 were predicted. Recombinant LysSA120 hydrolyzed cell walls were obtained from *S. aureus*, *S. epidermidis*, *S. haemolyticus*, *S. warneri*, *S. auricularis* and *S. saprophyticus*. It was shown that treatment of *S. aureus* planktonic cells with endolysin LysSA120 led to reduced viability. Furthermore, LysSA120 could hydrolyze mature biofilm formed by VRSA. The lytic spectrum and antibiofilm activity of LysSA120 warrant its further evaluation as an enzybiotic against drug-resistant staphylococcal infections.

## 1. Introduction

*Staphylococcus aureus* is one of the most widespread opportunistic bacteria. This microorganism can either be asymptomatically carried on human skin and mucous membranes or cause a range of clinical manifestations [1]. According to available data, approximately 30% of the human population is colonized by *S. aureus* [2,3]. This pathogen is the leading cause of skin and soft tissue infections (SSTIs), including folliculitis, furuncles, carbuncles, impetigo, staphylococcal scalded skin syndrome, and others [4]. Although most staphylococcal infections are not life-threatening, *S. aureus* can cause bacteremia, infective endocarditis, osteomyelitis, toxic shock syndrome, urinary tract infections, and other serious conditions [3,5,6].

*S. aureus* possesses several mechanisms that allow it to evade the host immune response, such as intracellular survival, protein A masking, biofilm formation, and the blockade of leukocyte chemotaxis. Of particular note, the ability of *S. aureus* strains to form biofilms and communicate within them via quorum sensing contributes to infections in prosthetic devices and implants [7,8,9].

The treatment of *S. aureus* infections is complicated by the emergence of strains with multidrug resistance, such as methicillin-resistant *S. aureus* (MRSA) [7]. It is worth noting that MRSA has retained its position in the high-priority pathogen category in the updated WHO Priority Pathogens List (2024), which identifies bacterial pathogens of global concern due to their antimicrobial resistance [7,10]. The diversity of intrinsic virulence factors contributes to the high recurrence rate of *S. aureus* infections. For example, among patients with urinary tract infections, up to 19% of initially cured patients experience recurrences [11,12]. Recurrent *S. aureus* infections pose a significant threat to individuals with various immunodeficiencies and metabolic disorders, such as diabetes mellitus, where *S. aureus* is the primary colonizer of diabetic foot ulcers, leading to lifelong recurrent SSTIs [7,13].

Consequently, the search for new antibiotics and approaches to combat multidrug-resistant pathogens, including MRSA, remains highly relevant today. Attempts are being made to develop a vaccine against *S. aureus* [14], but the efficacy of none of the candidate vaccines has been confirmed. A well-known and long-established approach is phage therapy, alongside which a new approach is actively emerging, involving the use of a novel class of antimicrobial agents termed enzybiotics [15,16,17,18]. This group includes bacteriophage and bacterial proteins that possess enzymatic activity promoting the degradation of bacterial polysaccharide polymers, such as bacterial cell wall peptidoglycan and capsular lipopolysaccharide [16].

Some of these proteins exhibit moderate activity and are required by bacteria for the restructuring of polysaccharide polymers during division (autolysins), or occasionally for interspecies competition (bacteriocins) [16,19,20]. Others are necessary for bacteriophage infection of the bacterium or for the subsequent release of viral progeny; these are virion-associated peptidoglycan hydrolases and endolysins, respectively. Endolysins are phage proteins that hydrolyze cell wall peptidoglycan, leading to bacterial destruction due to the disruption of osmotic pressure [21]. They often have a broader spectrum of susceptible bacteria compared to the parent bacteriophage, while retaining species and genus specificity.

Endolysins are classified based on their enzymatic activity into five different groups, depending on the specific peptidoglycan bond they target: lysozymes (N-acetylmuramidases), glycosidases (N-acetyl-β-D-glucosaminidases), amidases (N-acetylmuramoyl-L-alanine amidases), endopeptidases (L-alanoyl-D-glutamate endopeptidases), and lytic transglycosylases [22]. According to available data, peptidoglycan is a highly conserved target, making de novo resistance to endolysins rare [23]. Therefore, the combined use of several endolysins from different groups is a promising strategy for broadening the spectrum of activity when developing a combination product. Furthermore, the combined use of endolysins with antibiotics, bacteriophages, depolymerases, antimicrobial peptides, and other antibacterial agents is possible, which is also an undoubted advantage. Endolysins, and especially endolysins targeting Gram-positive bacteria (due to the absence of an outer membrane that restricts external access to the cell wall), represent a promising avenue for the development of new antibacterial drugs [21,24].

In the present study, the endolysin LysSA120, encoded by the *S. aureus* phage vB_SauP_120, was obtained and characterized. A comprehensive characterization of LysSA120 was performed, including in silico prediction of its domain organization and three-dimensional structure, as well as phylogenetic analysis aimed at assessing its evolutionary relationships with endolysins from other staphylococcal bacteriophages and identifying possible links between structural features and enzymatic activity. The anti-staphylococcal activity of LysSA120 was evaluated using a combination of in vitro assays. LysSA120 was shown to hydrolyze the cell walls of various staphylococcal species, including both coagulase-positive and coagulase-negative species. Furthermore, LysSA120 demonstrated the ability to suppress the growth of planktonic cultures of *S. aureus*. Particular emphasis was placed on the activity of this endolysin against biofilms formed by vancomycin-resistant *S. aureus*, given the well-established role of biofilms in infection persistence and reduced susceptibility to antimicrobial therapy.

## 2. Materials and Methods

### 2.1. Bacterial Strains and Growth Conditions

*Staphylococcus* and *Escherichia coli* strains were obtained from the Collection of Extremophile Microorganisms and Type Cultures (CEMTC) of ICBFM SB RAS. The *Staphylococcus* species was determined by sequencing the 16S rRNA gene fragment (~1400 bp). Antibiotic resistance has been previously tested using the disk-diffusion method (Oxoid™, Thermo Fisher Scientific, Waltham, MA, USA) in accordance with the EUCAST recommendations (version 8.0).

The following *E. coli* strains were used for cloning and protein expression: *E. coli* XL1 blue (F’:Tn10 proA+B+ lacIq Δ(lacZ)M15/recA1 endA1 gyrA96 (NalR) thi hsdR17 (rK– mK+) glnV44 relA1 lac) and *E. coli* M15 (pREP4) (F-, Φ80ΔlacM15, thi, lac-, mtl-, recA+, KmRK12 F- lambda- ilvG- rfb-50 rph-1) [malB+] K-12 (λS)).

Bacterial strains were cultivated in lysogeny broth (LB) media/agar (1.5% *w*/*v*) (10 g/L peptone, 5 g/L yeast extract, 10 g/L NaCl, pH: 7.0, Becton Dickinson, Franklin Lakes, NJ, USA). The planktonic bacteria cells were grown in an orbital shaker incubator with 150 rpm at 37 °C (Infors HT, Bottmingen, Switzerland). For biofilm production bacterial strains were cultivated in LB, containing 1% *v*/*v* glucose.

Bacteriophage *S. aureus* vB_SauP_120, previously isolated and characterized at the Institute of Chemical Biology and Fundamental Medicine (ICBFM SB RAS, Novosibirsk, Russia) under accession №120 (http://www.niboch.nsc.ru/doku.php/emtc_collection, accessed on 1 June 2026), was used in this study.

### 2.2. In Silico Identification and Structural Analysis of Putative Lysin

A Maximum Likelihood tree of 34 length-filtered (200–300 aa) BLASTp (https://blast.ncbi.nlm.nih.gov/Blast.cgi, accesion date 15 April 2026) homologs of LysSA120 was inferred in MEGA 11 from a MUSCLE alignment (default MUSCLE settings) under the WAG+G model with 1000 bootstrap replicates, SPR-level 3 heuristic, partial deletion (80% site coverage), and midpoint rooting.

The putative endolysin LysSA120 was analyzed using Protein Database from NCBI (https://www.ncbi.nlm.nih.gov/protein, accessed on 15 April 2026), InterProScan software package (https://www.ebi.ac.uk/interpro, accessed on 15 April 2026), HHpred tool (https://toolkit.tuebingen.mpg.de/tools/hhpred, accessed on 21 January 2026), and COFACTOR function prediction server (https://aideepmed.com/COFACTOR/, accessed on 19 May 2025) [25]. To calculate solubility of LysSte134_1 and LysSte134_2, Protein-sol [26] (https://protein-sol.manchester.ac.uk, accessed on 18 August 2025) and SoluProt [27] (https://loschmidt.chemi.muni.cz/soluprot, accessed on 18 August 2025) were used. The assumed three-dimensional (3D) structures of the endolysin LysStA120 was predicted using AlphaFold2 (https://colab.research.google.com/github/sokrypton/ColabFold/blob/main/AlphaFold2.ipynb, accessed on 21 May 2025) [28] and AlphaFold3 (https://alphafoldserver.com/, accessed on 19 January 2026) [29]. Structural visualization was performed using UCSF Chimera molecular visualizer, version 1.15 [30].

### 2.3. Cloning, Expression and Purification of Recombinant Lysins

The gene encoding endolysin LysSA120 was amplified by PCR using the genomic DNA of bacteriophage vB_SauP_120 as a template and the primers endolysin_56_SA120_1U29 and endolysin_56_SA120_728L23 (Table 1). Plasmid pQE-60 was digested with *Nco*I and *Bam*HI restriction endonucleases (SibEnzyme, Novosibirsk, Russia). The PCR products were also digested with *Nco*I/*Bam*HI and ligated into the linearized vector using T4 DNA ligase (SibEnzyme, Novosibirsk, Russia). The resulting recombinant plasmid pQE-60/LysSA120 was used to transform competent *Escherichia coli* XL1-Blue cells. Transformants were grown overnight on LB agar supplemented with 50 μg/mL ampicillin at 37 °C. *E. coli* clones carrying the expression plasmid were identified by colony PCR using primers pQE60-SeqU and pQE60-SeqL (Table 1). The correctness of the insert was confirmed by Sanger sequencing on a 3500 Genetic Analyzer (Thermo Fisher Scientific, Waltham, MA, USA).

For optimization of recombinant LysSA120 expression, *E. coli* M15 strain containing the pQE-60/LysSA120 plasmid was used. An overnight culture of *E. coli* M15-pQE-60/LysSA120 was grown in 5 mL of LB containing 50 μg/mL ampicillin at 37 °C. For protein production, the overnight culture was diluted 1:100 into 100 mL of fresh LB medium with 50 μg/mL ampicillin and incubated at 37 °C until the optical density at 600 nm (OD_600_) reached 0.6–0.7. Expression was then induced using various concentrations of isopropyl β-D-1-thiogalactopyranoside (IPTG): 25, 50, 100, 250, and 500 µM. Induction was carried out at temperatures of 10, 20, 30, and 37 °C for 4, 8, and 16 h with shaking at 180 rpm. Cells were harvested by centrifugation (5 min, 6000× *g*), resuspended in 50 mM Tris-HCl, pH 8.0, and disrupted by sonication using a Sonopuls HD 2070 sonicator (Bandelin, Mecklenburg-Vorpommern, Germany). Lysates were fractionated into soluble (cytoplasmic) and insoluble (inclusion body) fractions by centrifugation (15 min, 14,000× *g*). Expression level and localization of recombinant LysSA120 were analyzed by 12.5% SDS-PAGE.

Recombinant LysSA120 was purified from the inclusion body fraction under denaturing conditions using Ni-NTA agarose (Qiagen, Venlo, The Netherlands) following the manufacturer’s instructions. *E. coli* M15-pQE-60/LysSA120 cells were harvested after induction and fractionated as described above. The inclusion body pellet was resuspended in a buffer containing 2 M urea (2 M urea, 50 mM NaH_2_PO_4_, 300 mM NaCl, 5 mM Tris-HCl, pH 8.0), sonicated, and the insoluble inclusion bodies were pelleted by centrifugation (20 min, 14,000× *g*). The pellet was dissolved in Buffer A (6 M urea, 50 mM NaH_2_PO_4_, 300 mM NaCl, 5 mM Tris-HCl, pH 8.0), sonicated again, and the solubilized inclusion bodies were separated from insoluble debris by centrifugation (30 min, 14,000× *g*). The resulting supernatant was loaded onto a Ni-NTA agarose column. LysSA120 was eluted with Buffer B (6 M urea, 50 mM NaH_2_PO_4_, 300 mM NaCl, 5 mM Tris-HCl, pH 8.0, 500 mM imidazole).

Fractions containing the endolysin were sequentially dialyzed against buffer D (50 mM Tris-HCl, 150 mM NaCl, 200 mM imidazole, 200 mM sucrose, pH 7.5) containing stepwise reduced urea concentrations 2 M, 1 M and 0 M, respectively. Each dialysis step was performed at 5–6 °C for 6–8 h with stirring on a magnetic stirrer. The refolded protein was concentrated using an Amicon Ultra-4 centrifugal filter unit (Millipore, Burlington, MA, USA) with a 10 kDa molecular weight cutoff. The buffer was exchanged for storage buffer (S-buffer: 50 mM Tris-HCl, pH 7.5, 300 mM NaCl) by repeated dialysis. Protein purity was quantified using a GelDoc Go gel documentation system (Bio-Rad, Hercules, CA, USA). Protein concentration was determined using a Qubit Protein Assay Kit (Thermo Fisher Scientific, Waltham, MA, USA) on a Qubit 4 fluorometer (Thermo Fisher Scientific, Waltham, MA, USA).

### 2.4. Zymography with Peptidoglycans from Staphylococcal Cells

Peptidoglycan was isolated from the cell walls of *Staphylococcus* spp. using previously described protocol [31] with modifications. Briefly, an overnight bacterial culture was diluted 1:500 in 1 L of LB medium and grown at 37 °C with shaking at 150 rpm until the optical density at 600 nm (OD_600_) reached 1.0–1.5. Cells were harvested by centrifugation for 10 min at 6000× *g* or 8000× *g* (the centrifugal force was adjusted according to the *Staphylococcus* species). The pellet was resuspended in 10 mL of 4 M LiCl, incubated in a boiling water bath for 20 min, and pelleted again by centrifugation. The resulting precipitate was resuspended in deionized water and disrupted by ultrasonication for 25 min at 35% amplitude using a Sonopuls HD 2070 homogenizer (Bandelin, Berlin, Germany). The homogenate was centrifuged at 12,000× *g* for 12 min. The pellet was then resuspended in 12 mL of 4% sodium dodecyl sulfate (SDS), boiled for 20 min, and collected by centrifugation (12,000× *g*, 12 min). The precipitate was washed with 12 mL of 1 M NaCl, followed by centrifugation under the same conditions; this washing step was repeated until the pellet became colorless. The final colorless pellet was suspended in 1–2 mL of deionized water, re-centrifuged, and dissolved in 2 mL of deionized water containing 0.02% sodium azide (NaN_3_). The purified peptidoglycan was stored at 4 °C.

Peptidoglycan concentration was measured using a SmartSpec Plus spectrophotometer (Bio-Rad, Hercules, CA, USA). An absorbance of 1.0 at 540 nm corresponded approximately to 1 mg/mL of peptidoglycan.

To evaluate the hydrolytic activity of recombinant endolysins, proteins were separated by SDS-PAGE in gels containing 0.1 mg/mL purified peptidoglycan as a substrate. Following electrophoresis, the gel was rinsed several times gently with deionized water and then incubated in a renaturation buffer consisting of 25 mM Tris-HCl (pH 7.2) supplemented with 1% Triton X-100. After 1 h of incubation at 37 °C, the gel was washed several times with deionized water and finally stained with a 1% methylene blue solution.

### 2.5. Biofilm Assay

Staphylococcal biofilms were prepared using previously described protocol [32]. Briefly, bacterial cells (2 × 10^8^ CFU) were suspended in 200 µL of sterile 0.9% NaCl, combined with 10 mL of LB medium, and transferred into a Petri dish containing a pre-sterilized glass coverslip. The culture was incubated at 37 °C for 5 days to allow biofilm development. On day 5, mature biofilms were gently washed with buffer to remove residual medium, after which 50 µL of recombinant endolysin LysSA120 at a concentration of 0.1 mg/mL was applied. Biofilm formation and the effect of LysSA120 were then assessed by light microscopy using a Zeiss Axio Imager A2 microscope (Carl Zeiss, Oberkochen, Germany).

Mature biofilms were then exposed to 25 µg/mL of purified endolysin diluted in phosphate-buffered saline (PBS). As a negative control, biofilms were treated with PBS alone without the enzyme. Following treatment, the samples were incubated at 37 °C and subsequently stained with 0.1% methyl violet (crystal violet) to visualize residual biofilm biomass.

### 2.6. Statistical Analyses

The statistical analysis was carried out by one-way analysis of variance (ANOVA) using Statistica 10 software (StatSoft. Inc., Tulsa, OK, USA). The differences between the groups were considered significant at *p* < 0.05.

## 3. Results

### 3.1. Phylogenetic Analysis of the St_134 Proteins

**Figure 1:**
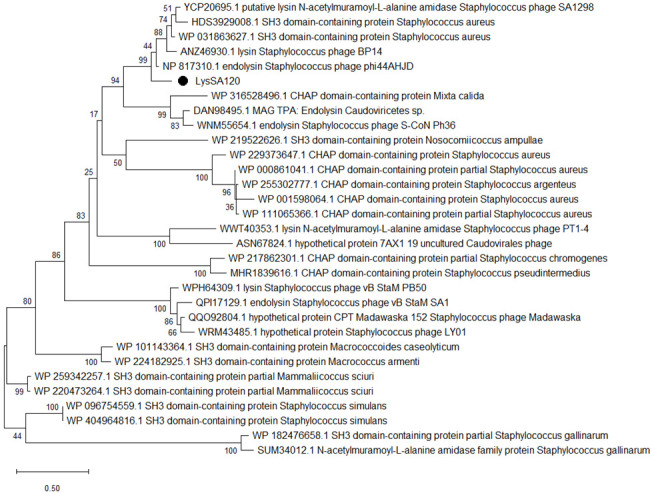
Maximum likelihood phylogenetic tree of LysSA120 and 30 BLASTp homologs (200–300 aa), inferred under the WAG+G model with 1000 bootstrap replicates in MEGA 11 and midpoint-rooted. Numbers at nodes indicate bootstrap support. The scale bar represents 0.5 amino acid substitutions per site. The position of LysSA120 is indicated by a filled black circle.

Phylogenetic placement of Lys SA 120. Lys SA 120 grouped within a strongly supported clade (bootstrap = 99) with the endolysins of Staphylococcus phages phi44AHJD (NP_817310.1) and BP14 (ANZ46930.1), nested in a broader monophyletic cluster (bootstrap = 95) that included the lysin of phage SA1298 (YCP20695.1) and two prophage-derived chromosomal homologs from *S. aureus* (HDS3929008.1, WP_031863627.1; bootstrap = 87). All immediate neighbors belong to the genus Rosenblumvirus (family Rountreeviridae)—small lytic staphylococcal podoviruses with ~17–18 kbp genomes encoding a distinctive lysis module of two compact peptidoglycan hydrolases. The size (250 aa) and clustering pattern identify Lys SA 120 as a CHAP-type peptidase lysin characteristic of Rosenblumvirus-like staphylococcal phages (Figure 1).

### 3.2. In Silico Characterization and Phylogenetic Analysis of LysSA120

Endolysin LysSA120 consists of 250 amino acids (aa). According to computational data, LysSA120 has a predicted molecular weight of approximately 28 kDa and a pI of 8.91 (Protein Sol, Job ID: 413071d16465e2e3a200). Two domains were identified in the structure of the endolysin. The N-terminal domain, based on the NCBI database (https://www.ncbi.nlm.nih.gov/protein, accessed on 25 September 2025) and InterProScan software (https://www.ebi.ac.uk/interpro, accessed on 20 September 2025), corresponds to the so-called CHAP domains (Cysteine, Histidine-dependent Amidohydrolases/Peptidase, IPR007921, pfam05257). The second, C-terminal domain corresponds to SH3_5 domains (pfam08460, IPR003646). In the endolysin sequence, the CHAP domain is located between aa 25 and 107, and the SH3_5 domain between aa 164 and 229. These domains are commonly found in staphylococcal bacteriophage endolysins and are also present in peptidoglycan hydrolases and peptidoglycan-recognizing proteins from various organisms.

The solubility of the recombinant LysSA120 protein was assessed using the bioinformatics tools SoluProt and Protein-sol. According to Protein-sol calculations, the solubility coefficient was 0.475, which is sufficient for obtaining soluble protein in *E. coli* cells given a threshold value of <0.45. In contrast, analysis using SoluProt yielded a solubility coefficient of 0.459 for LysSA120, which is insufficient for its production in *E. coli* (required value > 0.5).

**Figure 2:**
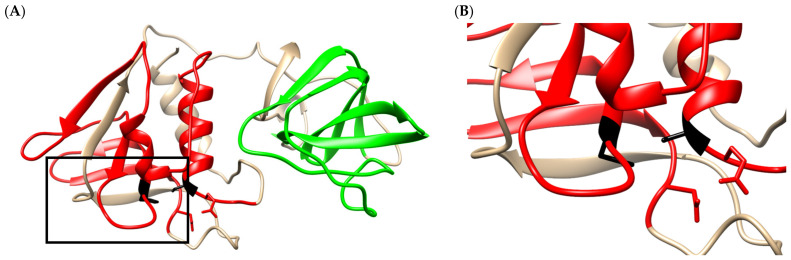
(**A**) Ribbon representation of the predicted 3D structure of the endolysin LysSA120; the CHAP domain and SH3_5 domain is shown in red and green, respectively. (**B**) Ribbon representation of the putative catalytic site of LysSA120; residues Cys 29 and His 89 are colored black and shown in stick representation. The molecular coordinates of the predicted 3D structure of LysSA120 were rendered using the UCSF Chimera molecular visualizer, version 1.15.

Modeling revealed that LysSA120 consists of two compact domains connected by a flexible linker. The N-terminal CHAP domain exhibits a compact and stable structure containing six β-strands and two α-helices, which are linked by short disordered linkers. The SH3_5 domain, which contains two tightly packed antiparallel β-sheets formed by seven β-strands, is attached to the CHAP domain via a flexible linker. Analysis using the COFACTOR metaserver (accessed on 10 February 2026), a tool for predicting protein–ligand binding sites, revealed structural similarity to proteins with hydrolase activity. Using COFACTOR, the amino acid residues forming the putative binding site of the CHAP domain were predicted to be Gln 28, Cys 29, Tyr 87, His 89, and Asn 107. Residues Cys 29 and His89 likely constitute the catalytic center of the enzyme (Figure 2).

### 3.3. Cloning, Production and Purification of LysSA120

**Figure 3:**
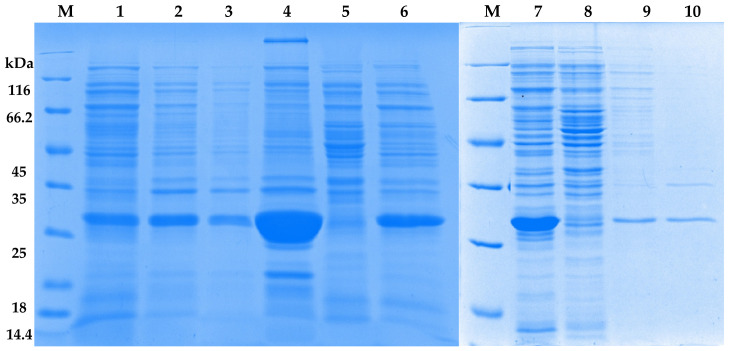
SDS-PAGE analysis of recombinant LysSA120 expression and purification. Lane M–protein ladder Standard (Catalog no.: 26610; Thermo Fisher Scientific, Hercules, CA, USA); lane 1—inclusion bodies of *E. coli* M15 pQE-60/LysSA120 in buffer A containing 4 M urea; lane 2—inclusion bodies in buffer A with 2 M urea; lane 3—inclusion bodies in buffer A with 4 M urea; lane 4—inclusion bodies in buffer A with 6 M urea; lane 5—soluble cytoplasmic fraction of *E. coli* M15 pQE-60/LysSA120; lane 6—lysate of *E. coli* M15 pQE-60/LysSA120 cells producing recombinant LysSA120; lane 7—flow-through fraction after Ni-NTA agarose chromatography; lane 8—eluate from Ni-NTA agarose after washing with buffer A; lane 9—eluate from Ni-NTA agarose after elution with buffer B; lane 10—purified recombinant LysSA120 after dialysis.

The gene encoding the endolysin LysSA120 was inserted into the expression plasmid pQE-60. To produce the recombinant endolysin LysSA120, the constructed plasmid pQE-60-LysSA120 was used to transform *E. coli* M15 cells. Various growth conditions were tested to increase the solubility of the recombinant endolysin. The localization and yield of LysSA120 were evaluated using PAGE (Figure 3). LysSA120 was not detected in the soluble fraction. The protein was purified from inclusion bodies using Ni-NTA agarose and subsequently refolded. Its molecular weight was approximately 28 kDa, which corresponded to the predicted value (Figure 3). The purity of LysSA120 after chromatography was evaluated by PAGE and determined to be ~95% (Figure 3). The purified LysSA120 was stored at a concentration of more than 0.5 mg/mL in buffer S (50 mM Tris-HCl, pH 7.5, 300 mM NaCl) at 4 °C.

### 3.4. Enzymatic Activity of LysSA120

**Figure 4:**
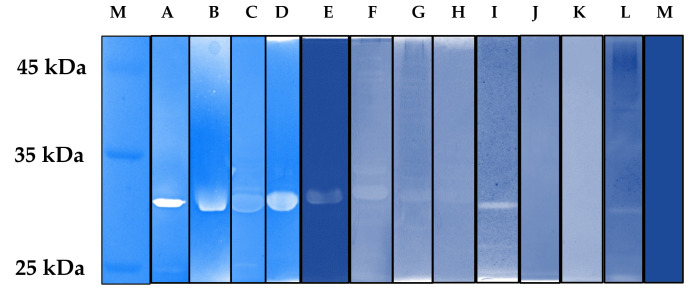
Zymographic analysis of recombinant LysSA120. PAGE (12.5%) containing 0.5 mg of peptidoglycan from *S. aureus* CEMTC 1685 (A), *S. epidermidis* CEMTC 2058 (B), *S. aureus* CEMTC 675 (C), *S. haemolyticus* CEMTC 3413 (D), *S. aureus* CEMTC 628 (E), *S. epidermidis* CEMTC 1827 (F), *S. warneri* CEMTC 1255 (G), *S. haemolyticus* CEMTC 1657 (H), *S. auricularis* CEMTC 2738 (I), *S. felis* CEMTC 2998 (J), *S. hominis* CEMTC 1702 (K), *S. saprophyticus* CEMTC 7267 (L), and *S. simulans* CEMTC 1728 stained with methylene blue. (M)– protein ladder Precision Plus Protein™ Standards (Bio-Rad, Hercules, CA, USA). During zymography, purified bacterial cell walls were incorporated into the separating PAGE gel during casting, and each gel contained a different type of cell wall. Therefore, it was not possible to assess endolysin activity against multiple distinct cell walls on a single gel, and lanes A–M had to be compiled from several gels into one composite image.

The lytic activity of endolysin LysSA120 was assessed by zymographic assay using cell wall peptidoglycans of various *Staphylococcus* strains as substrates. It was shown that LysSA120 hydrolyzed peptidoglycan from the cell wall of *S. aureus* CEMTC 1685 (the host strain of phage SA120), as well as from *S. aureus* CEMTC 628 and *S. aureus* CEMTC 675 (Figure 4). *S. aureus* CEMTC 675 is resistant to cefoxitin, gentamicin, erythromycin, and clindamycin (multidrug-resistant, MDR), whereas *S. aureus* CEMTC 1685 is resistant to lincomycin and vancomycin (VRSA).

In addition, LysSA120 was able to digest peptidoglycans from coagulase-negative staphylococci, namely *S. epidermidis* CEMTC 1827 (MDR), *S. epidermidis* CEMTC 2043 (β-lactam resistant), *S. haemolyticus* CEMTC 1657 (weak activity, MDR), *S. haemolyticus* CEMTC 3413 (MDR), *S. warneri* CEMTC 1255 (amikacin-resistant), *S. auricularis* CEMTC 2738 (clindamycin-resistant), and *S. saprophyticus* CEMTC 7267 (Figure 4, Table S1). However, it could not cleave peptidoglycans isolated from the cell wall of *S. hominis* CEMTC 1702, *S. felis* CEMTC 2998, or *S. simulans* CEMTC 1728. The obtained results demonstrate that LysSA120 is capable of hydrolyzing peptidoglycan from the cell wall of various pathogenic *Staphylococcus* strains, regardless of their antibiotic resistance profiles.

### 3.5. Anti-Staphylococcal Activity of LysSA120

**Figure 5:**
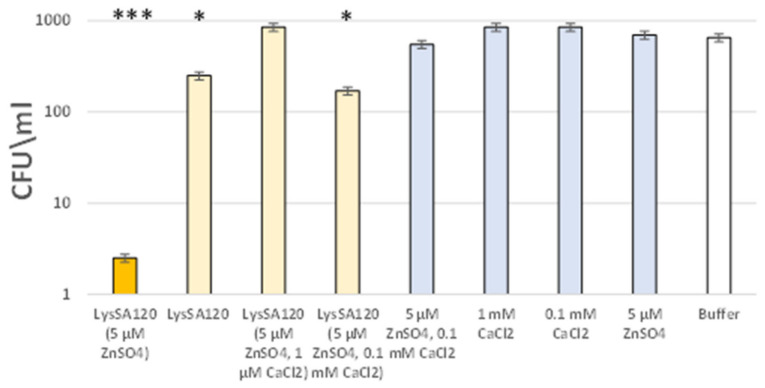
Antibacterial activity of LysSA120 against *S. aureus* CEMTC 1685. LysSA120 (10 µg/mL) was added to staphylococcal cells with or without CaCl_2_ or ZnCl_2_, and the resulting suspensions were incubated for two hours prior to viable colony counting. Cell cultures containing only CaCl_2_ or ZnCl_2_ were used as controls. Experiments were performed in triplicate. Statistical significance was assessed by ANOVA (*** *p* < 0.001, * *p* < 0.05).

The antibacterial activity of LysSA120 was assessed against a planktonic culture of *S. aureus* CEMTC 1685. It was demonstrated that treatment of *S. aureus* CEMTC 1685 cells with LysSA120 (10 µg/mL) resulted in a 3-fold decrease in CFU (Figure 5). Subsequently, the effect of different concentrations of bivalent ions (Ca^2+^ and Zn^2+^) on the efficacy of LysSA120 was studied. When *S. aureus* CEMTC 1685 cells were treated with LysSA120 supplemented with 5 µM ZnCl_2_, the lytic activity of LysSA120 against this strain increased up to 100-fold (Figure 5). Notably, the addition of Ca^2+^ completely inhibited the hydrolytic activity of LysSA120. When Ca^2+^ and Zn^2+^ were added together, the hydrolytic activity of LysSA120 was also completely inhibited. The addition of Ca^2+^ and Zn^2+^ without LysSA120 did not affect the viability of *S. aureus* CEMTC 1685 cells.

### 3.6. Biofilm Disruption Activity of LysSte134_1

The activity of recombinant LysSA120 was also evaluated using a biofilm formed by *S. aureus* strain CEMTC 1685. The mature biofilms were incubated with 5 µg of the recombinant endolysin LysSA120 for three hours at 37 °C, then fixed, stained, and examined using light microscopy. Sterile storage buffer was added as a control.

**Figure 6:**
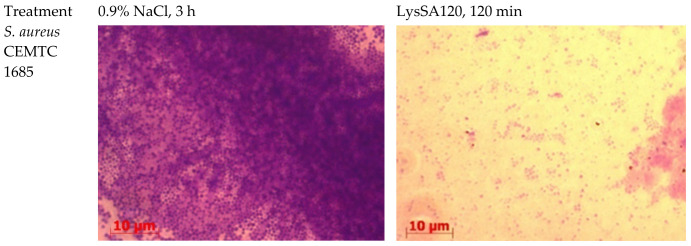
LysSA120 activity against biofilms formed by vancomycin-resistant *S. aureus* CEMTC 1685. Bacterial cells were stained with methylene blue.

The control biofilm exhibited an extensive matrix densely filled with bacteria stained with methyl violet. In contrast, the biofilm treated with LysSA120 showed small fragments of the biofilm matrix with no significant number of bacteria within the matrix, as well as a markedly lower overall number of remaining bacteria compared to the control sample (Figure 6).

## 4. Discussion

The spread of antibiotic-resistant bacteria necessitates the development of novel antimicrobial agents as alternatives to conventional antibiotics. Numerous alternative strategies are currently being explored, including quorum sensing inhibition [33], suppression of virulence factors [34], antimicrobial monoclonal antibodies [35], bacteriophages [36], and phage endolysins [37]. Among these approaches, the use of endolysins appears particularly promising because resistance to them is rarely developed, and the wide distribution of bacteriophages in nature ensures their diversity [38].

The endolysin LysSA120 consists of two domains: an N-terminal catalytic CHAP domain and a C-terminal cell wall-binding SH3b domain, which represents the typical architecture of endolysins from Gram-positive bacteriophages [21]. Phylogenetic analysis places LysSA120 within a well-supported clade containing endolysins from staphylococcal phages phi44AHJD and BP14 (bootstrap = 99), as well as within a broader cluster encompassing the genus Rosenblumvirus (bootstrap = 95). The two closest homologous sequences correspond to SH3-containing proteins, likely from temperate bacteriophages, encoded chromosomally in sequenced *S. aureus* isolates rather than in active phage genomes. The AlphaFold-predicted model and binding site predictions using COFACTOR converge on Cys29 and His89 as the catalytic residues of the CHAP domain of LysSA120, while Gln28, Tyr87, and Asn107 form the binding center.

Treatment with 10 μg/mL LysSA120 resulted in an approximately three-fold reduction in viable *S. aureus* CEMTC 1685 cells compared to the control, and the addition of 5 μM ZnCl_2_ enhanced this activity by two orders of magnitude. In contrast, 5 μM CaCl_2_ completely abolished enzyme activity, both when used alone and in combination with zinc. This distinguishes LysSA120 from all staphylococcal CHAP enzymes characterized to date.

The parental bacteriophage is capable of lysing only *S. aureus* strains, whereas LysSA120 hydrolyzes cell walls from *S. aureus*, *S. epidermidis*, *S. haemolyticus*, *S. warneri*, *S. auricularis*, and *S. saprophyticus*. These findings confirm that bacteriophages are generally species-specific and have a limited range of host strains, while endolysins exhibit a significantly broader lytic spectrum, including the ability to hydrolyze cell walls of bacteria that are not hosts of the original phage [39,40]. One reason for the narrow host range of bacteriophages is the presence of various intracellular defense mechanisms against phage infection. To achieve cell lysis, a bacteriophage must enter the host cell, replicate, and only after progeny maturation does lysis occur from within via phage endolysins. At any stage of this cycle, the bacterium can evade lysis through its defense systems. In contrast, an endolysin lyses the cell from the outside, and its enzymatic activity typically targets a conserved chemical bond in the peptidoglycan. Consequently, bacteria have very limited opportunities to develop resistance to endolysins [41].

The activity of LysSA120 against multidrug-resistant strains was retained across all susceptible bacterial species, including cefoxitin- and gentamicin-resistant *S. aureus* CEMTC 675 and vancomycin-resistant *S. aureus* CEMTC 1685. This indicates that LysSA120 acts independently of the antibiotic resistance phenotype of the target strain. The lack of activity against peptidoglycans from *S. hominis*, *S. felis*, and *S. simulans* suggests species-specific differences in cell wall composition, which varies among different *Staphylococcus* species [42].

*S. aureus* is well known for its ability to form biofilms, which poses a serious challenge for the eradication of this pathogen in clinical settings and often leads to antibiotic treatment failure even against susceptible strains [5,43]. Endolysins exhibit exceptional efficacy in eliminating biofilm-associated infections [44]. Our results demonstrate that LysSA120 displays pronounced activity against mature biofilms formed by vancomycin-resistant *S. aureus*. After biofilm disruption by LysSA120, conventional antimicrobial agents and host innate immunity can subsequently eliminate the remaining planktonic bacteria [45]. These findings suggest that LysSA120 may be considered a promising candidate for the development of a biofilm-removing agent. Nevertheless, the overall safety and therapeutic efficacy of LysSA120 require further comprehensive investigation.

## Figures and Tables

**Table 1:** Primers used for cloning and sequencing.

Primer Title	Primer Sequence
endolysin_56_SA120_U	5′-GGGAACCATGGGAATGAAATCACAACAACAAGCAAAAGAATG-3′
endolysin_56_SA120_L	5′-GGGATGGATCCTGAGAACACCCCCCACGGTACAC-3′
pQE60-SeqU	5′-GATTCAATTGTGAGCGGATAAC-3′
pQE60_SeqL	5′-ATCCAGATGGAGTTCTGAGGTC-3′

## Data Availability

Data are contained within the article and Supplementary Materials.

## Supplementary Materials

The following supporting information can be downloaded at: https://www.mdpi.com/article/10.3390/v18060654/s1, Table S1. Characteristics of *Staphylococcus* strains.

## Abbreviations

The following abbreviations are used in this manuscript:

MRSA	Methicillin-resistant *S. aureus*
LB	Luria–Bertani
CFU	Colony-forming units
MDR	Multidrug-resistant
SDS-PAGE	Sodium dodecyl sulfate–polyacrylamide gel electrophoresis
PFU	Plaque-forming units
OD	Optical density
PCR	Polymerase chain reaction
IPTG	Isopropyl β-D-1-thiogalactopyranoside
EDTA	Ethylenediaminetetraacetic acid
ANOVA	Analysis of variance
NCBI	National Center for Biotechnology Information
PDB	Protein Data Bank
ICBFM SB RAS	Institute of Chemical Biology and Fundamental Medicine, Siberian Branch of the Russian Academy of Sciences
MDR	Multidrug resistance
R	Resistance
S	Sensitive
XDR	Extensively Drug-Resistant
