# Supplementary material for: Cloning and Characterization of an Endolysin LysSA120 as a Potential Staphylococcus Biofilm-Removing Agent
Source: Viruses. 2026 Jun 9;18(6):654. doi: 10.3390/v18060654 (PMC13307668; doi:10.3390/v18060654)
Supplement: Supplementary file 1 [file viruses-18-00654-s001.zip › viruses-4333746-supplementary.pdf]

Table S1. Characteristics of *Staphylococcus* strains.

**CEMTC**	**Sample source**	**β-lactams**	**Aminoglycosides**	**Macrolides**	**Lincosamides**	**Fluoroquinolones**	**Chloramphenicol**	**Glycopeptides**	**Other antibiotics**	**Antibiotic resistance**
S. aureus										
**628**	exudate of a purulent fistula									S
**675**	bedsore	FOX	CN	E	DA					MDR
**1685**	throat swab							VA		R
S. epidermidis										
**1827**	diabetic foot ulcer	FOX	AK, CN	E	DA					MDR
**2058**	fluid from the joint	P								R
S. haemolyticus										
**1657**	wound	FOX	CN	E						MDR
**3413**	oral mucosa	FOX	AK, CN	E						MDR
**S. auricularis**										
**2738**	ear swab, otitis				DA					R
**S.saprophyticus**										
**7267**	oral cavity	P								R
**S. simulans**										
**1728**	diabetic foot ulcer									S
S.hominis										
**1702**	nasal swab									S
S. warneri										
**1255**	vagina, chronic inflammation		AK							R
S. felis										
**2998**	eye smear									S

Abbreviations: MDR – Multi Drug Resistance; R – Resistance; S – Sensitive; AK – Amikacin; CN – Gentamicin; DA – Clindamycin; FOX – Cefoxitin; E – Erytromycin; P – Penicillin; VA – Vancomycin.
